# O062: Contamination of umbilical catheters by Staphylococcus epidermidis in neonatology: is there a link with a change in the standard of care?

**DOI:** 10.1186/2047-2994-2-S1-O62

**Published:** 2013-06-20

**Authors:** I Soulake, A Gayet-Ageron, N Bochaton, S Touveneau, P Rimensberger, R Pfister, D Pittet, W Zingg

**Affiliations:** 1 Infection Control Programme, University of Geneva Hospitals (HUG), Geneva, Switzerland; 2 Enfant et adolescent, University of Geneva Hospitals (HUG), Geneva, Switzerland

## Introduction

The presence of *Staphylococcus epidermidis* (SCE) on umbilical venous (UVC) or artery catheters (UAC) suggests defects either in catheter care or in hand hygiene compliance.

## Objectives

The aim of this study was to assess colonization of UVC and UAC with SCE before and after care practice of such catheters was changed.

## Methods

This observational before-after study was conducted in the neonatology unit of HUG between January 2002 and December 2012. SCE-colonization rates before and after protocol change in August 2011 was compared by chi-square test and Poisson regression model. In the new protocol, UVC and UAC were not covered by a dressing but left at air. All neonates with the following risk factors were eligible: gestational age [GA] <32 weeks, birth weight <1500g, invasive device, surgery, use of parenteral nutrition, systemic antibiotics.

## Results

In total, 2832 neonates were analyzed. Mean birth weight (±SD) was 2179g (±970) and 213 neonates had GA < 32 weeks(7.5%). SCE colonization on UVC and UAC was 54/1070 (5.1%) and 16/435 (3.7%), respectively. Colonization on UVC was significantly higher after procedure change in the univariate (53.7/1000 catheter-days versus 9.6/1000; P<0.001) as well as in the multivariate analysis adjusting for GA, birth weight, and multiple pregnancy (IRR [95% CI]: 2.4 [1.4-4.3]; P=0.003). Colonization of UAC was significantly higher after procedure change in the univariate (43.5/1000 cathter-days versus 7.9/1000; P<0.001) as well as in the multivariate analysis (IRR [95%CI]: 5.0 [1.7-15.2]; P=0.004). No association was found for catheter-related bloodstream infection.

## Conclusion

Leaving umbilical catheters exposed to air rather than protected by a dressing may result in significant colonization with SCE. Larger studies must confirm our findings and test the hypothesis whether such practice promotes bloodstream infection.

## Disclosure of interest

None declared.

